# High-risk pregnancy and risk of breastfeeding failure

**DOI:** 10.1186/s42506-024-00172-w

**Published:** 2024-10-14

**Authors:** Eman S. Salama, Mostafa Hussein, Ahmed N. Fetih, Azza M. A. Abul-Fadl, Shimaa A. Elghazally

**Affiliations:** 1 Obstetrics & Gynecology Department, Faculty of Medicine, Merit University, Sohag Al Gadida City, Egypt; 2 Obstetrics & Gynecology Department, Faculty of Medicine, Assiut University, Assiut, Egypt; 3 Pediatrics Department, Faculty of Medicine, Certified Lactation Consultant, Benha University, Cairo, Egypt; 4 Public Health and Community Medicine Department, Faculty of Medicine, Assiut University, Assiut, Egypt

**Keywords:** Exclusive breastfeeding, Pregnancy, High-risk pregnancy, Noncommunicable diseases, Casarean section

## Abstract

**Background:**

There is growing evidence that supports the role of breastfeeding in reducing the burden of non-communicable diseases (NCDs). There are considerable gaps in breastfeeding outcomes in mothers with chronic diseases due to a lack of knowledge and support in the postpartum period. Mothers who have NCDs and pregnancy complications are at risk of breastfeeding failure.

**Aim:**

To compare breastfeeding outcomes in mothers with NCDs with healthy mothers and determine the underlying challenges that lead to poor outcomes.

**Methods:**

A prospective cohort study was conducted among 150 women (50 with high-risk pregnancies (HRP) and 100 with normal pregnancies (NP)). They were recruited from those attending the immunization and outpatient clinics at Sohag General Hospital. Mothers were recruited at 34 weeks gestation and were followed up at 2 weeks, 6 weeks, and 6 months after delivery. A pretested and validated questionnaire was used to collect detailed epidemiological, personal, health-related status, medications, hospitalizations, reproductive history, current delivery, and previous breastfeeding experiences. On follow-up they were assessed for breastfeeding practices, their health and health and growth of their children, and social support.

**Results:**

Delivery by cesarean section and postpartum bleeding were commoner among HRP patients. Initiation of breastfeeding in the 1st hour of delivery was significantly lower among women with HRP than those with normal pregnancies (48.0% versus 71.0%, *p* = 0.006). The most common reason for not initiating breastfeeding among the NP group was insufficient milk (34.5%), while in the HRP group, it was the mother’s illness (80.8%). Skin-to-skin contact with the baby after birth was significantly less practiced in the HRP than in the NP group (38.0% vs 64.0% at *p* = 0.003). Herbs (such as cumin, caraway, cinnamon, aniseed, and chamomile) were the most common pre-lacteal feeds offered (63.0% in NP vs 42.0% in HRP). Artificial milk was more used in HRP than NP (24.0% vs 4.0%). Breast engorgement was 3 times more common in the HRP compared to the NP group (61.5% vs19.6%). Stopping breastfeeding due to breast problems was 2.5 times higher in the HRP than in the NP group (38.5% vs. 15.2%, *p* = 0.003). Nipple fissures were twice as common among the NP than among the HRP group ((73.0%) vs. (38.5%), *p* = 0.026). Exclusive breastfeeding during the period of follow-up was lower in the HRP than in the NP group (40.0% vs 61.0%, *p* < 0.05) and formula feeding was twice as common in the HRP as in the NP group (34.0% vs. 18.0%, *p* = 0.015). Child illness was significantly higher among women with HRP than those with NP (66.0% vs 48.0%, *p* = 0.037).

**Conclusions:**

Women with HRP are at a high risk of poor breastfeeding outcomes with increased lactation problems and formula feeding rates. Encouraging women especially those with HRP to achieve optimal breastfeeding practices is a simple intervention that can be included in daily practice and may have a positive impact on mothers’ health.

## Introduction

High-risk pregnancy (HRP) is an increasing problem globally: populations in poor countries, as well as affluent ones, are at risk. HRPs are defined as pregnancies with preexisting or current conditions that put the mother or her fetus at higher risk for complications during pregnancy or after birth [1]. Apart from the risks of obstetric complications that can affect pregnancy and result in adverse outcomes for both the mother and the fetus, there are non-communicable diseases (NCDs) during pregnancy [2].

Breast milk contains all the nutrients an infant needs in the first 6 months of life and in addition, it has many health benefits for both the mother and infant. Exclusive breastfeeding means feeding the baby only breast milk, not any other foods or liquids (including infant formula or water), except for medications or vitamin and mineral supplements [3].

It was established that a decrease in breastfeeding practices is associated with an increase in the rate of NCDs such as diabetes, cardiovascular diseases, obesity, and autoimmune disorders [4]. Previous studies concluded that breastfeeding has been associated with a reduced risk of type 2 diabetes in both healthy mothers and mothers with gestational diabetes [5, 6]. Gunderson et al. found that longer breastfeeding duration was inversely associated with the risk of developing diabetes after delivery [7].

Women with high-risk pregnancies are less likely to exclusively breastfeed (EBF) their infants and may have shorter breastfeeding duration than those with normal pregnancies [8–10]. Several potential barriers to successful breastfeeding among those with HRP have been identified such as higher rates of cesarean section, premature delivery, premature rupture of membranes, maternal-infant separation, and delayed initiation of lactation [11] in addition to early introduction of formula feeding [12]. A longitudinal cohort study in Canada (*n* = 2706) of women who delivered a live-born infant between 2008 and 2010 found that prenatal medical risk severity and type were not significantly associated with breastfeeding initiation, except for pre-pregnancy risk type. Risk severity was associated with lower odds of breastfeeding to 4 months, 12 months, and earlier breastfeeding cessation. They found associations of shorter breastfeeding length across the first postpartum year for women with pre-pregnancy, current obstetric, and substance use risk types, but not past obstetric problems [13].

In Egypt, exclusive breastfeeding is common but not universal in very early infancy, and the proportion of exclusively breastfed drops rapidly among older infants [14]. Despite the importance of breastfeeding in reducing and controlling non-communicable diseases, women with certain health problems may be less likely to initiate and maintain breastfeeding. Few studies especially in Upper Egypt have investigated the association between NCDs and breastfeeding outcomes, leaving a gap in our understanding of the link between high-risk pregnancy and breastfeeding patterns. This study aims to compare the outcome of breastfeeding in mothers with NCDs with healthy mothers. Also to assess the challenges and barriers to breastfeeding initiation and continuation in HRP cases and the support needed to overcome these challenges.

## Methods

### Study design and setting

A prospective cohort study was conducted at Sohag General Hospital in Upper Egypt. The study populations were pregnant women of the reproductive age group (20–45 years old) who were followed up from the 34th week of pregnancy till delivery and at 2 weeks, 6 weeks, and 6 months after delivery. They were recruited from those attending the immunization and outpatient clinics at Sohag General Hospital. They were allocated into two groups: the normal pregnancy group, and the high-risk group.

### Participants

The inclusion criteria included women with a high-risk pregnancy if she has one or more of the following conditions: Diabetes mellitus and/or gestational diabetes mellitus, cardiac diseases (rheumatic and/or valvular heart diseases, essential hypertension and/or pregnancy with superimposed hypertension or preeclampsia), chronic chest disease (such as bronchial asthma and/or chronic TB infection), chronic hepatitis (such as viral hepatitis), neurologic disease for example (epilepsy), anemia (HB < 7 gm/dL) as indicated by the need for admission and blood transfusion and finally thyroid disease, either hypo-or hyperthyroidism. These risk factors were confirmed by full clinical examination and investigations. Women with none of the above risk factors were included in the study as the control group. Exclusion criteria included pregnant women < 34 weeks in the reproductive age group. The study was conducted over 24 months from September 2017 to September 2019.

### Sample

**Fig. 1:**
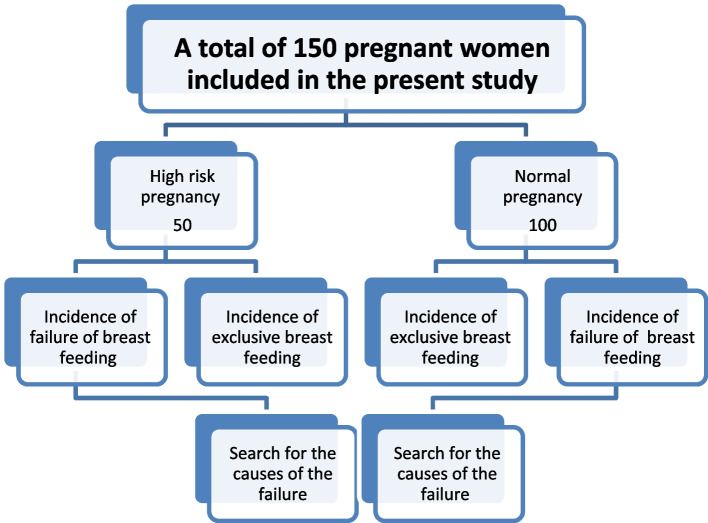
Study design diagram

The sample size was calculated using the Open EPI program with an expected response rate of exclusive breastfeeding (EBF) among the high-risk group of 72%, an expected response rate of EBF among the comparison group of 92%, and the ratio of the comparison group to cases 2:1. The total sample size at power 80 and confidence level 95 was 138 (46 high-risk pregnancies and 92 normal pregnancies). The actual collected sample was 150 (50 high-risk pregnancies and 100 normal pregnancies). A convenience sampling technique was applied in this study from both immunization and outpatient clinics until the sample size was reached (Fig. 1).

### Data collection methods

An interviewer-administered questionnaire was constructed after reviewing the related literature and was translated into the Arabic language by experts. The questionnaire was piloted on 20 women from the target group, who were excluded later from the study sample to determine acceptability and the clarity of questions, and to estimate the time needed for filling it; it was then modified accordingly. The reliability test was done by using Cronbach’s alpha. The reliability of the questionnaire used for internal consistency was 0.8. The questionnaire was used to obtain information on socio-demographic status, birth-related events, knowledge and practices related to breastfeeding during the first 6 months, sources of breastfeeding information, and family support. The questions on knowledge were in multiple-choice forms. Closed questions were used for the practices that support breastfeeding with explanations when necessary.

The mothers were recruited at 34 weeks of pregnancy and mothers with live births were followed up at three points of time: after delivery at 2 weeks, 6 weeks, and after 6 months. A personal follow-up card was given to every eligible woman which included all the planned times for the follow-up visits. The telephone was used to remind her to come or contact us with a response rate of 96%. They were assessed at those visits for breastfeeding practices, their general health, and the health and growth of their infants.

Growth assessment involves measuring a child's weight and height and comparing these measurements to growth standards to determine whether a child is growing normally or not**.** Measurements of children were taken by trained physicians who evaluated their measurements and plotting on their growth charts [15].

### Statistical analysis

The statistical Package for Social Sciences (IBM-SPSS), version 24 (May 2016); IBM, Chicago, USA, was used for statistical data analysis. The normality test was checked for continuous variables using the Shapiro–Wilk test, If the data showed a normal distribution, the mean and standard deviation (SD) were used. The Student *t*-test was used to compare the means between two groups, and the one-way analysis of variance (ANOVA) test was used to compare the means of more than two groups. If the data were not distributed normally, the Mann–Whitney *U* test was used, and variables were expressed as median values with interquartile range. For categorical variables, they were expressed as numbers and percentages. The chi-square test was used to compare proportions between groups. The p-value is considered significant if < 0.05.

### Ethical considerations

The study was approved by the Scientific Ethics Committee of the Faculty of Medicine, Assiut University. Confidentiality was assured, names did not appear on the questionnaire form and participants were identified by codes only.

## Results

Table 1 shows that the highest percentage (44.0%) of women with HRP had basic education, while around one-half of the women with NP had secondary education (52.0%) compared to 28.0% of HRP with a statistically significant difference between both groups (*P* = 0.013). HRP was more common among urban women compared to women with NP (16.0% vs 3.0%) with a statistically significant difference (*p* = 0.007).

**Table 1:** Maternal sociodemographic data of normal pregnancy and high-risk group, Sohag, Egypt, 2018–2019

Variable	Normal pregnancy *N* = 100		High-risk group *N* = 50		Total		*P* value
	No.	%	No.	%	No.	%	
Mother`s age (years)							
Mean ± SD	25.64 ± 5.29		26.46 ± 5.31		25.91 ± 5.29		0.373^#^
Father’s age: (years)							
Mean ± SD	32.19 ± 5.24		32.44 ± 5.50		32.27 ± 5.31		0.787^#^
Level of education							
Illiterate	11	11.0	8	16.0	19	12.7	
Basic education	22	22.0	22	44.0	44	29.3	0.013*
Secondary	52	52.0	14	28.0	66	44.0	
University	15	15.0	6	12.0	21	14.0	
Residence							
Rural	97	97.0	42	84.0	139	92.7	0.007*
Urban	3	3.0	8	16.0	11	7.3	
Occupation							
Housewife	91	91.0	47	94.0	138	92.0	0.751
Employee	9	9.0	3	6.0	12	8.0	

High-risk group: pregnant women with chronic co-morbidities

Normal pregnancy group: pregnant women with no chronic co-morbidities

^*^Statistically significant difference

^#^Student’s *t* test was used and in other variables, the chi squared test was used

Table 2 shows that antenatal care (ANC) visits were more frequent among the NP group than those of HRP (*p* = 0.004). The most common indication of previous delivery by cesarean section (CSD) among NP was contracted pelvis (14.6%, *p* = 0.017), while the most common indication of previous CSD among HRP was pre-eclampsia (18.9%, *p* = 0.002). Women with HRP delivered more frequently in governmental hospitals or tertiary levels (68.0%) compared to 51.0% in NP with a statistically significant difference (*p* = 0.048).

**Table 2:** Obstetric history between normal pregnancy and high-risk group

Obstetric history	Normal pregnancy *N* = 100		High-risk group *N* = 50		Total		*P* value
	No.	%	No.	%	No.	%	
Number of ANC visits							
Median (IQR)	6(4)		4(3)		5(4.5)		0.004*^#^
Previous deliveries							
Primigravida	27	27.0	16	32.0	43	28.7	0.523
Multipara	73	73.0	34	68.0	107	71.3	
No. of previous deliveries							
Median (IQR)	2(2)		2.5(2)		2(2)		0.168^#^
History of childhood illness	24	24.0	16	32.0	40	26.7	0.296
Mode of delivery							
NVD	50	50.0	11	22.0	61	40.7	0.001*
CS	50	50.0	39	78.0	89	59.3	
Number of previous CSs							
Median (IQR)	1(1)		1(1)		1(1)		0.591^#^
Indications of previous CS							
Breech	4	8.3	2	5.4	6	8.3	0.693
Cardiac	0	0.0	2	5.4	2	0.0	0.187
Contracted pelvis	7	14.6	0	0.0	7	14.6	0.017*
IUGR	1	2.1	0	0.0	1	2.1	1.000
Obstructed labor	14	29.2	5	13.5	19	29.2	0.086
Pre-eclampsia	0	0.0	7	18.9	7	0.0	0.002*
Previous CS	20	41.7	18	48.6	38	41.7	0.521
PROM	2	4.2	3	8.1	5	4.2	0.649
Place of delivery							
Governmental hospital	51	51.0	34	68.0	85	56.7	0.048*
Private center or clinic	49	49.0	16	32.0	65	43.3	

*PROM* premature rupture of membranes, *CS* cesarean section, *NVD* normal vaginal delivery, *BF* breastfeeding, *IUGR* intrauterine growth retardation

^*^Statistically significant difference

^#^The Mann–Whitney* U* test was used and in other variables chi-square test was used

**Fig. 2:**
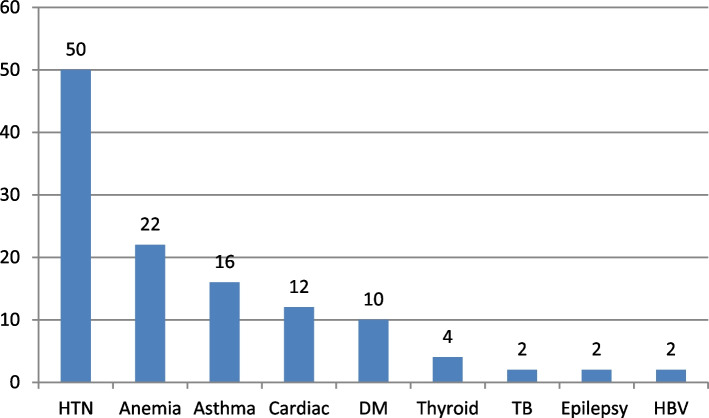
Medical disorders among high-risk pregnant women. HTN: hypertension, HBV: hepatitis B virus, TB: tuberculosis, DM: diabetes mellitus

Figure 2 illustrates the common conditions that were prevalent in the HRP group. These included hypertension (HTN) (50.0%), anemia (22.0%), chronic obstructive lung disease (16.0%), cardiac condition (12.0%), diabetes mellitus (DM) (10.0%), thyroid disorders (4.0%), tuberculosis (TB) (2.0%), epilepsy (2.0%), and hepatic disease due to hepatitis B virus (HBV) (2.0%).

**Fig. 3:**
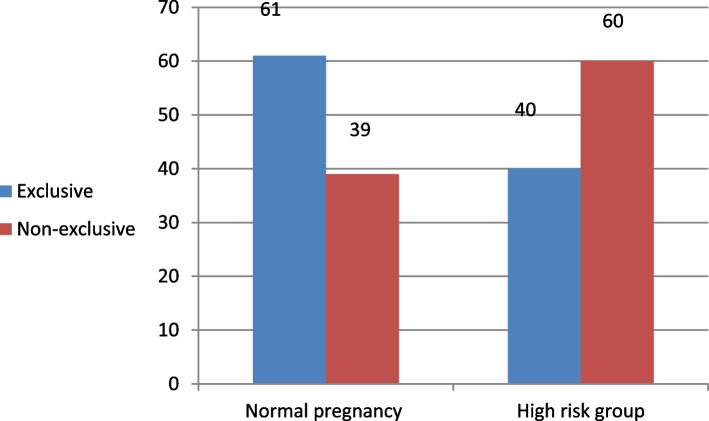
Exclusive breastfeeding patterns in normal pregnancy and high-risk groups

Table 3 shows that more mothers of the NP group were assisted in holding their baby skin-to-skin (STS) after birth than those of HRP (64.0% vs 38.0%). This was statistically significant (*p* = 0.003). Also, the initiation of breastfeeding within the first hour of delivery was significantly higher among women with NP than those with HRP (71.0% vs 48.0%, *p* = 0.006). The most common reason for not initiating breastfeeding among women with NP was insufficient milk (34.5%), whereas the most common reason for not initiating breastfeeding among those with HRP was mother illness (80.8%, *p* = 0.001 and *p* = 0.0001 respectively). The most common type of pre-lacteal feeds offered to babies among women with NP was herbs (63.0%), while artificial milk was high among those with HRP (24.0%) compared to only 4.0% among NP women (*p* = 0.015 and *p* = 0.0001 respectively). Women with NP were allowed rooming in with their babies more frequently than those of HRP (84.0% vs 62.0%, *p* = 0.003). Women of the HRP felt they had less social support than women with NP (62.0% vs 80.0%) with a statistically significant difference (*p* = 0.018). Exclusive breastfeeding rates (EBF) were significantly higher in the NP than in the HRP (61.0% vs 40.0%, *p* < 0.05) (Fig. 3).

**Table 3:** Practices of breastfeeding among normal pregnancy and high-risk group

Practices of breastfeeding	Normal pregnancy *N* = 100		High-risk group *N* = 50		Total		*P* value
	No.	%	No.	%	No.	%	
Initiation of breastfeeding in the 1st hour of delivery	71	71.0	24	48.0	95	63.3	0.006*
Reason for not initiating breastfeeding:^a^							
Colostrum is not good	5	17.2	2	7.7	7	12.7	0.426
No milk	10	34.5	0	0.0	10	18.2	0.001*
Mother was sick (medical problem)	3	10.3	21	80.8	24	43.6	0.0001*
Baby was sick	4	13.8	1	3.8	5	9.1	0.355
Baby was taken away from me	9	31.0	7	26.9	16	29.1	0.737
Skin-to-skin contact with baby after Birth	64	64.0	19	38.0	83	55.3	0.003*
Offering pre-lacteal feeds to baby	69	69.0	35	70.0	104	69.3	0.900
Type of feeding:^a^							
^b^Herbs	63	63.0	21	42.0	84	56.0	0.015*
Artificial milk	4	4.0	12	24.0	16	10.7	0.0001*
Glucose	2	2.0	3	6.0	5	3.3	0.334
Water	2	2.0	2	4.0	4	2.7	0.601
Date	2	2.0	2	4.0	4	2.7	0.601
Practiced rooming-in	84	84.0	31	62.0	115	76.7	0.003*
Return of menstruation							
Yes	63	63.0	36	72.0	99	66.0	0.273
LAM (EBF)	37	37.0	14	28.0	51	34.0	
Use of other contraceptive methods	57	57.0	29	58.0	86	57.3	0.907
Social support for breastfeeding	80	80.0	31	62.0	111	74.0	0.018*

^*^Statistically significant difference

^a^Chi-square test was used

^b^Such as cumin, caraway, cinnamon, aniseed, and chamomile

Breast engorgement was more frequent among those with HRP (61.5%), while nipple fissures occurred more frequently among those with NP (73.9%) (*p* = 0.0001 and *p* = 0.003, respectively). Breastfeeding cessation was higher among those with HRP than those with NP (38.5% vs 15.2%, *p* = 0.026). Dealing with breast problems by soothing agents was higher among those with normal pregnancies (17.4%) with a statistically significant difference between both groups (*p* = 0.044). Puerperal complications were almost twice as common in the HRP as in the NP (42.0% vs 27.0%) but the difference was not significant (*p* = 0.063) (Table 4).

**Table 4:** Comparison of the health problems during breastfeeding between women with normal pregnancy and the high-risk group

Health problems	Normal pregnancy *N* = 100		High-risk group *N* = 50		Total		*P* value
	No.	%	No.	%	No.	%	
History of development of breast problems	46	46.0	26	52.0	72	48.0	0.488
Type of breast problems							
Breast engorgement	9	19.6	16	61.5	25	34.7	0.0001*
Mastitis	3	6.5	0	0.0	3	4.2	0.549
Nipple fissures	34	73.9	10	38.5	44	61.1	0.003*
Management of breastfeeding problems							
Expressing breast milk	12	26.1	11	42.3	23	31.9	0.156
Stopping breastfeeding	7	15.2	10	38.5	17	23.6	0.026*
Giving bottles with an artificial nipple	19	41.3	5	19.2	24	33.3	0.056
Resorting to soothing agents such as pacifiers	8	17.4	0	0.0	8	11.1	0.044*
Puerperal bleeding	36	36.0	24	48.0	60	40.0	0.157
Puerperal complications	27	27.0	21	42.0	48	32.0	0.063

^*^Statistically significant difference, chi-square test was used

History of child illness was significantly higher among women with HRP than those of NP (66.0% vs 48.0%, *p* = 0.037). The most common baby-related reason for stopping breastfeeding among NP was the refusal of the baby to breastfeed (60.9%) compared to 28.6% of HRP women with a statistically significant difference between the two groups (*p* = 0.032) while the most common reason for stopping breastfeeding among those with HRP was the admission of the baby to the neonatal intensive care unit (NICU) (33.3%compared only to 8.7% of NP women), however, the difference between the groups was not statistically significant (*p* > 0.05).

Starting complementary food at a younger age (< 2 months) was higher among those with HRP than those with NP (36.0% vs 23.0%). Starting in older ages (4–6 months and > 6 months) was more frequent among those with normal pregnancies (46.0% and 23.0% respectively) than high-risk group (42.0% and 12.0% respectively) but the difference was not significant (*p* > 0.05) (Table 5).

**Table 5:** Comparison of feeding practices, baby health and growth, and mother satisfaction between normal pregnancy and high-risk group

Feeding practices, baby health and growth, and mother satisfaction	Normal pregnancy *N* = 100		High-risk group *N* = 50		Total		*P* value
	No.	%	No.	%	No.	%	
History of child illness	48	48.0	33	66.0	81	54.0	0.037*
Continuation of breastfeeding even with the baby’s illness	26	26.0	11	33.3	37	45.7	0.064
Expression of breast milk for the baby when mothers are away	10	10.0	4	8.0	14	9.3	0.775
Age of start complementary food							
< 2 months	23	23.0	18	36.0	41	27.3	
2–4 months	8	8.0	5	10.0	13	8.7	0.219
4–6 months	46	46.0	21	42.0	67	44.7	
> 6 months	23	23.0	6	12.0	29	19.3	
Reasons for cessation of breastfeeding^a^							
Child refused by himself	14	60.9	6	28.6	20	45.5	0.032*
Getting pregnant	4	17.4	3	14.3	7	15.9	1.000
Child is not feeding well	4	17.4	1	4.8	5	11.4	0.348
Admission to the NICU	2	8.7	7	33.3	9	20.5	0.064
Having medical problems during pregnancy	3	13.0	7	33.3	10	22.7	0.155
Normal baby growth (by growth charts)	71	71.0	35	70.0	106	70.7	0.899
Mother’s satisfaction with baby growth	71	71.0	35	70.0	106	70.7	0.899

^*^Statistically significant difference, chi-square test was used

^a^Percentages do not sum up to 100% because of multiple responses

Table 6 shows a higher level of knowledge about breastfeeding among those with exclusive breastfeeding than those with non-exclusive breastfeeding. Knowledge about the benefits of breastfeeding on diabetic women especially its role in reducing blood glucose, losing the gained weight, and preventing prediabetes and diabetes was significantly higher among mothers practicing exclusive breastfeeding.

**Table 6:** Knowledge about breastfeeding between mothers practicing exclusive and non-exclusive breastfeeding

Knowledge about breastfeeding	Exclusive BF (*n* = 81)		Non-exclusive BF (*n* = 69)		*P* value
	No.	%	No.	%	
Advantages of breastfeeding:^a^					
It is nutritious for the baby	79	97.5	65	94.2	0.414
Protects the baby from infections	80	98.8	63	91.3	0.049*
Mother baby bonding	81	100.0	66	95.7	0.095
Cheap and available	80	98.8	59	85.5	0.002*
Contraception method	30	37.0	27	39.1	0.792
Maintains mother's body weight	37	45.7	28	40.6	0.530
Prevents maternal breast cancer	52	64.2	38	55.1	0.256
Benefits of exclusive breastfeeding on diabetic women:^a^					
Reducing blood glucose	41	50.6	20	29.0	0.007*
Losing weight gained	55	67.9	36	52.2	0.049*
Preventing prediabetes and diabetes	38	46.9	16	23.2	0.003*
Providing the best food for the newborn	80	98.8	66	95.7	0.334
Enhancing the immunity of the newborn	81	100.0	69	100.0	–
Proper techniques of breastfeeding:^a^					
Use both breasts at each feeding	76	93.8	50	72.5	0.0001*
Breastfeed day and night	81	100.0	52	75.4	0.0001*
Good attachment	78	96.3	55	79.7	0.001*
Use of EBM when the mother is away	6	7.4	7	10.1	0.553
Definition of EBF:^a^					
To give only breast milk and medicines if indicated	41	50.6	38	55.1	0.586
To give breast milk and water	26	32.1	40	58.0	0.001*
Recommended duration of EBF					
1 month	0	0.0	7	10.1	0.004*
2 months	2	2.5	4	5.8	0.414
3 months	2	2.5	7	10.1	0.081
4 months	8	9.9	22	31.9	0.001*
5 months	7	8.6	5	7.2	0.754
6 months	52	64.2	24	34.8	0.0001*
8 months	6	7.4	0	0.0	0.031*
1 year	4	4.9	0	0.0	0.125
Dangers of bottle feeding					
Can cause diarrhea	72	88.9	59	85.5	0.535
Nipple confusion	58	71.6	43	62.3	0.227

^*^Statistically significant difference, chi-square test was used

^a^Multiple responses

## Discussion

High-risk pregnancy (HRP) is a significant problem in Egypt. Exclusive breastfeeding (EBF) is recommended for the first 6 months of life. This can support the lactational amenorrhea method (LAM) of contraception which depends on EBF and high frequency of breastfeeding, especially at night. This study showed lower rates of LAM and early return of menstruation among the HRP group and this may be explained by low EBF rates among them. In this study, EBF was more frequent among those with normal pregnancies (NP) (61%) than those with HRP (40%). An increase in the rate of NCDs such as diabetes and cardiovascular diseases (CVDs) is likely associated with a decrease in the practice of breastfeeding [16]. In Egypt, the national rates of EBF have been shown by the Egypt Family Health Survey in 2022 to decline progressively over the first months of life to reach 20.7% at 4–5 months [14].

Upper Egypt usually has the lowest rates of EBF due to the high offering of fluids because of the common misconception that babies need more fluids in hot weather. Despite this, EBF was lower in HRP indicating that these women were probably introducing early weaning foods and milk formula. They thought that their disease state necessitated stopping or reducing breastfeeding because of their condition. This was practiced by many HRP women irrespective of being highly educated and of urban residence. Moreover, women with HRP were less likely to have regular ANC and were more likely to end up with CSD and delivery in a tertiary-level hospital than NP, especially in cases with pre-eclampsia.

The most common conditions in the HRP were HTN (50.0%) followed by chronic anemia (22%). Hypertensive disorders of pregnancy occur in approximately 7%–10% of pregnancies and are associated with adverse maternal cardiovascular health outcomes across the lifespan. In contrast, breastfeeding has been associated with a reduction in cardiovascular risk factors in a dose-dependent manner [17].

A meta-analysis of 6 studies including more than 20,000 mothers showed that breastfeeding was associated with a relative risk reduction of 30% for diabetes and 13% for hypertension among studied participants and these findings suggest that breastfeeding is associated with long-term health benefits, including a reduction in the risk of future maternal chronic diseases [18].

A study conducted in Canada (2022) on breastfeeding women with hypertension showed that hypertensive disorders of pregnancy were associated with an increase in the odds of non-exclusive breastfeeding at 4 months postpartum. They had significantly higher odds of reporting insufficient milk supply and lower odds of breast and/or nipple pain compared with those without hypertensive disorders of pregnancy [17]. A prospective study (2023) showed that 30.6% of mothers with chronic conditions were at higher risk of early cessation of breastfeeding in the first 6 months [19]. A protocol shows that a randomized behavioral trial will be conducted among mothers with hypertensive disorders during pregnancy to assess the effect of a breastfeeding self-efficacy-based intervention which will be delivered by a trained lactation consultant in the hospital on postpartum blood pressure and breastfeeding continuation [20].

In this study, a considerable percentage of our cases of HRP were attributable to DM. Breastfeeding plays an important role in reducing blood glucose levels and preventing or at least delaying the development of type 2 diabetes among women with histories of gestational diabetes [21]. Prolactin production during breastfeeding stimulates insulin secretion from beta cells and produces serotonin. This hormone is an antioxidant and helps in the reduction of oxidative stress which makes the mother’s beta-pancreatic cells healthier [22].

A cohort study showed that gestational DM in primiparous women did not affect their duration of breastfeeding. They emphasized that the positive health effects of breastfeeding in preventing overweight and obesity are needed to minimize the risk of type 2 diabetes for themselves and their offspring [23]. Similarly, a study in Australia reported that Indigenous women with type 2 diabetes had lower odds for EBF at discharge (adjusted OR 0.4) than women with no hyperglycemia in pregnancy but at 6 weeks and 6 months there was no significant difference between the groups. They concluded that Indigenous women were more likely to predominantly breastfeed at 6 weeks across all levels of hyperglycemia [24].

Our results showed that antenatal care (ANC) visits were significantly less frequent among the HRP group. An observational trial among Scandinavian women found that antenatal breast milk expression (ABE) was feasible and increased the rates of EBF in women with DM. The researchers showed that implementing a structured ABE guideline for women with medically treated diabetes was feasible. Furthermore, the intervention was associated with a high level of satisfaction among study participants. No obvious side effects were observed, and breastfeeding rates at discharge and 6–8 weeks after delivery were higher than in comparable studies [25].

The results of the present study show that the initiation of breastfeeding within the first hour of delivery was significantly lower among women with HRP. The underlying causes of delayed initiation of breastfeeding in DM may be because maternal diabetes and obesity can delay lactogenesis. Matias et al. 2014 reported that one-third of women with GDM experienced delayed onset of lactogenesis and that maternal obesity, insulin treatment, and suboptimal in-hospital breastfeeding were the key risk factors for early breastfeeding failure [26]. A review of the beneficial effects of breastfeeding and gestational diabetes concluded that efforts should be made to support women with DM to breastfeed especially since breastfeeding was found to be protective against the development of DM in infants later in life and their mothers [27].

Moreover, we have shown that women with HRP were more at risk of breast problems, especially breast engorgement, and for dealing with these problems breastfeeding cessation was a common practice. Other researchers have shown that breastfeeding difficulties are the most common reason for breastfeeding cessation, particularly in the early postpartum and cause mothers to be less likely to breastfeed a future child [28].

Cesarean section delivery (CSD) was more common in our group of mothers with HRP compared to the NVD. Over one-half of women in Egypt are exposed to CSD. CSD was 66.4% in Upper Egypt (UE) compared to 78.5% in Lower Egypt and 75% in urban governorates. In UE it was higher in urban areas compared to rural areas (76.2% vs 63.3%) [14]. Furthermore in this study delayed breastfeeding initiation, shorter duration of breastfeeding, and higher rates of non-exclusive breastfeeding among HRP have been accentuated by the finding of higher CSD in the HRP. This has also been mentioned by other studies [29, 30]. Cesarean surgery can place high stress on both the mother and infant, and post-operative recovery is often characterized by maternal pain, limited mobility, and separation from the infant to encourage mothers to rest and heal [31]. One study in Canada showed that CSD was associated with higher odds of low milk supply and infant behavior/health difficulties than women who deliver vaginally [32]. A systemic review concluded that CSD is associated with long-term risks for mothers, babies, and subsequent pregnancies [33].

Some of the HRP cases were due to bronchial asthma or respiratory diseases. Literature shows that breastfeeding for more than 6 months was associated with a reduced risk of wheeze, bronchiolitis, and wheeze-related healthcare utilization in infants at risk due to maternal asthma. Notably, breastfeeding for shorter durations was associated with a reduced risk of healthcare utilization compared with none. The researchers suggest that larger cohorts are needed to further examine the impact of breastfeeding exposure on respiratory health in infants exposed to maternal asthma [34].

There are considerable gaps in breastfeeding outcomes in mothers with chronic diseases due to a lack of knowledge and support in the postpartum period [35]. Evidence supports a correlation between maternal chronic conditions and adverse perinatal outcomes, including increased risk for preeclampsia, cesarean section, preterm birth, and admission to the neonatal intensive care unit (NICU). However, there is a knowledge gap about the management of these women during lactation. The present study showed a higher level of knowledge about breastfeeding among those practicing exclusive breastfeeding than those who did not. It was concluded from an Egyptian study conducted by Emara et al., 2021 that mothers with good knowledge about the proper practices of breastfeeding adhered more to exclusive breastfeeding (OR 2.51) and they emphasized the importance of proper health education and sufficient practical training the mothers about proper breastfeeding practice to raise exclusive breastfeeding rate [36].

### Study limitations

The study had some limitations as recall bias; some of the mothers were not able to recall all the details of their practices in the first 6 months. Being more informed, mothers who come to the hospital might give the desired answers even if they do not practice. Sample selection was obtained via a convenience-based non-probability technique which may result in a lack of representation of all classes and limit its generalizability.

## Conclusion

Women with HRP were at a high risk of poor breastfeeding outcomes with increased lactation problems and formula feeding rates. In HRP, such as women with hypertensive disorders, DM, bronchial asthma, and other chronic diseases especially when there is underlying obesity, anemia, and poverty breastfeeding may offer a safe and feasible low-cost intervention to reduce the burden of NCDs for these women in their children which is interpreted in high-cost savings at the national level. Support should be provided to instruct and encourage breastfeeding, especially for women with HRP.

## Data Availability

The datasets used and/or analyzed during the current study are available from the corresponding author on reasonable request.

## Abbreviations

NCD: Non-communicable disease
HRP: High-risk pregnancies
NP: Normal pregnancies
CVD: Cardiovascular disease
IBM-SPSS: Statistical Package for Social Sciences
PROM: Premature rupture of membranes
CSD: Cesarean section delivery
NVD: Normal vaginal delivery
EBF: Exclusive breastfeeding
LAM: Lactational amenorrhea method
NICU: Neonatal intensive care unit
